# Fibrinogen is related to long-term mortality in Chinese patients with acute coronary syndrome but failed to enhance the prognostic value of the GRACE score

**DOI:** 10.18632/oncotarget.15094

**Published:** 2017-02-04

**Authors:** Yong Peng, Tian-Li Xia, Yi-Ming Li, Fang-Yang Huang, Hua Chai, Peng-Ju Wang, Wei Liu, Chen Zhang, Xiao-Bo Pu, Shi-Jian Chen, Mao Chen, De-Jia Huang

**Affiliations:** ^1^ Department of Cardiology, West China Hospital, Sichuan University, Chengdu, China; ^2^ West China School of Medicine, Sichuan University, Chengdu, China

**Keywords:** acute coronary syndrome, fibrinogen, GRACE risk score, prognosis, Pathology Section

## Abstract

Fibrinogen (Fib) is considered to be a potential risk factor for the prognosis of patients with acute coronary syndrome (ACS), but it is unclear whether Fib level have synergistic effects to enhance the prognostic value of the GRACE score in patients with ACS. A retrospective analysis was conducted from a single registered database. 2253 consecutive patients with ACS confirmed by coronary angiography were enrolled and were grouped into 3 categories by the tertiles of admission plasma Fib levels. The end points were all-cause mortality and cardiac mortality. The mean follow-up time was 27.2 ± 13.1 months and death events occurred in 223 cases and cardiac death events occurred in 130 cases. Cumulative survival curves indicated that the risk of all-cause death increased with increasing Fib level (mortality rates for Tertile 1 *vs*. Tertile 2 *vs*. Tertile 3 = 6.6% vs. 10.8 %vs. 12.3%, *p* < 0.001). Cox multivariate regression analysis indicated that compared with other traditional risk factors, plasma Fib level is independently correlated with all-cause death (HR 1.33, 95% CI 1.04-1.70). However, incorporating elevated Fib level into the GRACE model did not significantly increase the predictive value of the GRACE score; for instance, AUC only increased from 0.703 to 0.713 (*p* = 0.765). In conclusion, Fib level at admission was independently associated with death risk among Chinese patients with ACS. However, the incorporation of Fib level at admission into the GRACE score did not improve this score’s predictive value for death risk among these patients.

## INTRODUCTION

Fibrinogen (Fib) is an important factor in the process of blood coagulation, serves as a main component in thrombosis, and is regarded as an important inflammatory marker [1]. The correlations among Fib, coronary heart disease (CHD), and cardiovascular events have long drawn research attention. A series of studies have found that an increase in Fib level is not only an independent risk factor for atherosclerosis and CHD [2, 3] but also *plays* an important *role in predicting* cardiovascular events and related deaths in patients with CHD [4, 5].

Acute coronary syndrome (ACS) is a manifestation of serious and potentially life-threatening CHD, including ST-segment elevation myocardial infarction (STEMI), non-STEMI, and unstable angina pectoris. The clinical prognoses of patients with ACS vary greatly. Therefore, accurate early risk stratification will help to determine correct and timely medical decisions for patients with ACS and will thereby improve these patients’ prognoses [6, 7]. The global registry of acute coronary events (GRACE) risk score is a risk-stratification tool recommended by guidelines and has good predictive ability in risk assessments for in-hospital mortality and long-term mortality among patients with ACS.[8–10] The most important clinical and laboratorial variables used by this risk scoring system include demographics, medical history and presentation characteristics at admission, such as heart rate, systolic blood pressure, serum creatinine, and troponin. As a result, this score is widely used in clinical practice. However, the GRACE score considers only two biomarkers, creatinine and troponin [9, 11], and does not incorporate certain biomarkers with important prognostic value. Recent studies have found that indicators of inflammation, such as C-reactive protein (CRP) and neutrophil count, enhance the predictive value of the GRACE score [12, 13]. However, it is unclear whether Fib has similar effects.

The purposes of this study are (1) to investigate the relationship between Fib level at admission and prognosis among Chinese patients with ACS and (2) to analyse whether Fib level at admission and GRACE score have synergistic effects with respect to predicting the prognoses of patients with ACS.

## RESULTS

**Figure 1:**
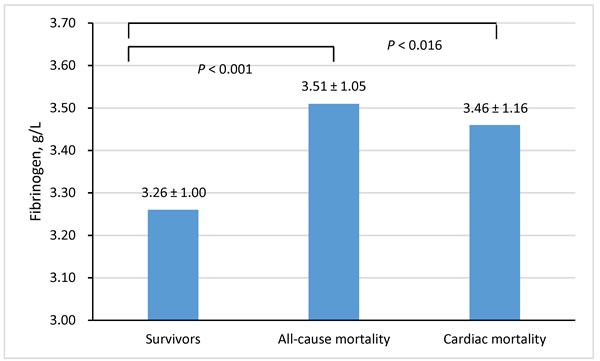
Fibrinogen levels across the survivors and non-survivors with all-cause mortality and cardiac mortality

A total of 2253 patients with CHD were included in this study; the distribution of baseline data is presented in Table 1. The average age of the patients was 64.7 (± 10.6) years, and the percentage of male patients was 78.7 %. Overall, patients died at an older age with a higher rate of diabetes, a faster heart rate at admission, more severe heart failure, and coronary artery disease. The average Fib level was 3.27 ± 1.01g/L. Fib level at admission was higher among patients who later died due to any cause and among patients who experienced cardiac death than among surviving patients (Figure 1). The tertile cut-off points for Fib level at admission were 2.79 g/L and 3.57 g/L. Using these cut-off points, patients were divided into three groups: Tertile 1 (< 2.79 g/L), Tertile 2 (2.79 to 3.57 g/L), and Tertile 3 (≥ 3.57 g/L).

**Table 1:** Baseline characteristics of the study population

Characteristics	Overall patients	All-cause death	Survivors	*p* value
No. of patients	*n* = 2253	*n* = 223	*n* = 2030	
Age, yrs	64.7 (10.6)	70.8 (8.8)	64.0 (10.5)	<0.001
Gender, men, n (%)	1774 (78.7)	164 (73.5)	1610 (79.3)	0.046
**Medical history**				
Pre-hypertension, n (%)	1225 (54.4)	127 (57.0)	1098 (54.1)	0.415
Pre-diabetes mellitus, n (%)	495 (22.1)	68 (30.8)	427 (21.1)	0.001
**At admission**				
Systolic blood pressure, mm Hg	130.3 (22.3)	128.1 (27.2)	130.6 (21.7)	0.130
Diastolic blood pressure, mm Hg	76.3 (12.9)	73.4 (13.8)	76.6 (12.7)	0.001
Heart rate, beats/min	75.3 (25.9)	80.6 (18.5)	74.7 (26.5)	0.001
Killip classification ≥ II, n (%)	382 (17.0)	83 (37.2)	299 (14.7)	<0.001
GRACE score	92.85 (26.01)	109.93 (24.84)	91.00 (25.46)	<0.001
**Laboratory values**				
Serum creatinine, μmol/L	94.3(51.2)	112.8 (74.9)	92.3 (47.5)	<0.001
Blood glucose, mmol/L	7.3 (3.5)	8.6 (4.4)	7.2 (3.4)	<0.001
Total cholesterol, mmol/L	4.1 (1.1)	4.0 (1.1)	4.1 (1.1)	0.208
**Severity of CAD**				
Left main artery, n (%)	228 (10.1)	40 (17.9)	188 (9.3)	<0.001
Three vessel diseases, n (%)	645 (28.6)	106 (47.5)	539 (26.6)	<0.001
**Discharge medications**				
Aspirin, n (%)	2084 (92.5)	149 (66.8)	1935 (95.3)	<0.001
Clopidogrel, n (%)	2073 (92.0)	154 (69.1)	1919 (94.5)	<0.001
Statin, n (%)	2029 (90.1)	150 (67.3)	1879 (92.6)	<0.001
ACE inhibitors or ARBs, n (%)	1270 (56.4)	91 (40.8)	1179 (58.1)	<0.001
Beta-receptor blockers, n (%)	1468 (65.2)	97 (43.5)	1371 (67.5)	<0.001

Data are expressed as means ± SD or counts and percentages, as appropriate.

Abbreviations: CAD, coronary artery disease; ACE, angiotensin-converting enzyme; ARBs, angiotensin-receptor blockers.

**Figure 2:**
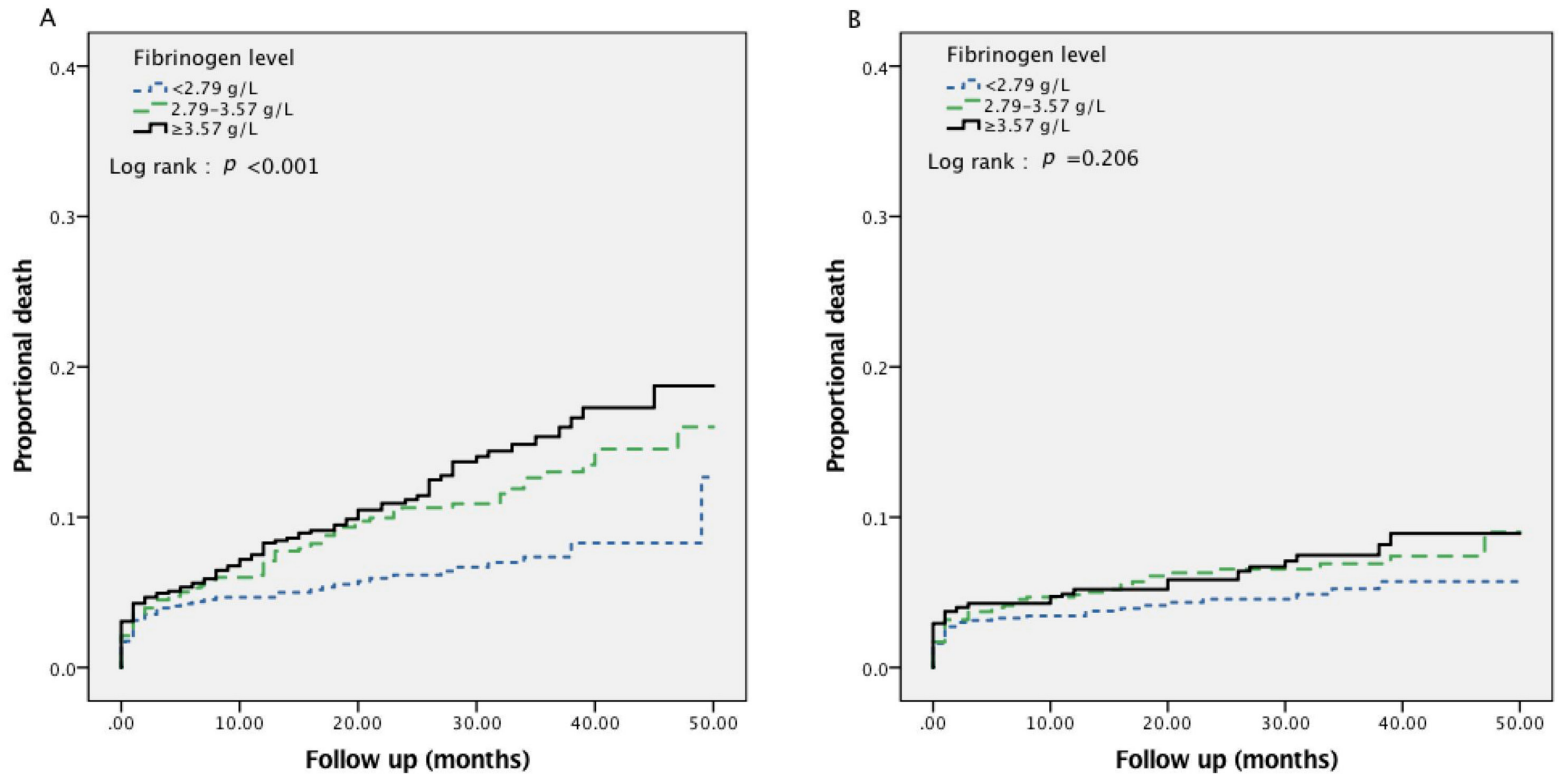
Kaplan-Meier survival curve for all-cause mortality (**panel A**) and cardiac mortality (**panel B**) in total 2253 patients with ACS according to fibrinogen levels.

In total, 2253 patients were followed up for a mean period of 27.2 ± 13.1 months; during this time, 223 patients died (mortality rate: 9.9%), including 130 cases (cardiac mortality rate: 5.8%) of cardiac death. Cumulative survival curves grouped by Fib level indicated that the risk of all-cause death increased with increasing Fib level (mortality rates for Tertile 1 *vs*. Tertile 2 *vs*. Tertile 3 = 6.6% *vs*. 10.8 %*vs*. 12.3%, *p* < 0.001) (Figure 2, panel A). Similar trends were observed for cardiovascular death, although the differences between tertiles were not statistically significant (cardiac mortality rate, Tertile 1 *vs*. Tertile 2 *vs*. Tertile 3 = 4.6% *vs*. 6.3% *vs*. 6.4%, *p* = 0.206) (Figure 2, panel B).

Univariate analysis revealed that the highest event rates were observed in the highest tertile, which exhibited a 1.96-fold [95% confidence interval (CI) 1.39-2.77] increase in all-cause mortality and 1.47-fold (95% CI 1.03-2.10) increase in cardiac mortality relative to the lowest tertile. After adjusting for confounders, Fib continued to provide relevant risk information for all-cause mortality [hazard ratio (HR) 1.83, 95% CI 1.09-3.08] (Table 2). Cox multivariate regression analysis indicated that compared with other traditional risk factors, plasma Fib level is independently correlated with all-cause death (HR 1.33, 95% CI 1.04-1.70) (Table 3).

**Table 2:** Hazard ratios for all-cause mortality and cardiac mortality across tertiles of fibrinogen

	All-cause mortality		Cardiac mortality	
Fibrinogen, g/L	HR (95% CI) Unadjusted	HR (95% CI) Adjusted*	HR (95% CI) Unadjusted	HR (95% CI) Adjusted*
Tertile 1	1	1	1	1
Tertile 2	1.66 (1.17-2.37)	1.62 (0.94-2.79)	1.39 (0.97-1.99)	1.40 (0.73-2.69)
Tertile 3	1.96 (1.39-2.77)	1.83 (1.09-3.08)	1.47(1.03-2.10)	1.28 (0.67-2.45)

^*^Risk factors adjustment included age, sex, medical history (pre-hypertension and pre-diabetes mellitus), admission examination (systolic blood pressure, diastolic blood pressure, and heart rate), heart function (Killip level), admission lab test (blood glucose, total cholesterol, and serum creatinine), severity of CAD (left main artery and three vessel diseases), discharge medication (aspirin, clopidogrel, statin, ACE inhibitors or ARBs and beta-receptor blockers).

Abbreviations: HR, hazard ratio; CI, confidence interval; CAD, coronary artery disease; ACE, angiotensin-converting enzyme; ARBs, angiotensin-receptor blockers.

**Table 3:** Results of multivariate Cox proportional-hazards model regarding follow-up events

Characteristics	All-cause Mortality		Cardiac Mortality	
	HR (95% CI)	*p*	HR (95% CI)	*p*
Fibrinogen*	1.33 (1.04-1.70)	0.025	1.12 (0.82-1.53)	0.466
Age†	1.63 (1.30-2.04)	<0.001	1.40 (1.06-1.83)	0.017
Men	1.34 (0.84-2.14)	0.220	1.65 (0.90-2.99)	0.103
Heart rate	1.00 (0.99-1.00)	0.882	1.00 (0.99-1.01)	0.958
Systolic blood pressure	1.00 (0.98-1.01)	0.602	0.99 (0.98-1.01)	0.368
Serum creatine‡	1.06 (0.96-1.17)	0.257	1.01 (0.90-1.14)	0.880

^*^grouped by the tertiles; † for 10-year increase; ‡ for each 20 μmol/L increase.

Risk factors adjustment included age, sex, medical history (pre-hypertension and pre-diabetes mellitus), admission examination (systolic blood pressure, diastolic blood pressure, and heart rate), heart function (Killip level), admission lab test (blood glucose, total cholesterol, and serum creatinine), severity of CAD (left main artery and three vessel diseases), discharge medication (aspirin, clopidogrel, statin, ACE inhibitors or ARBs and beta-receptor blockers)

Abbreviations: HR, hazard ratio; CI, confidence interval; CAD, coronary artery disease; ACE, angiotensin-converting enzyme; ARBs, angiotensin-receptor blockers.

**Figure 3:**
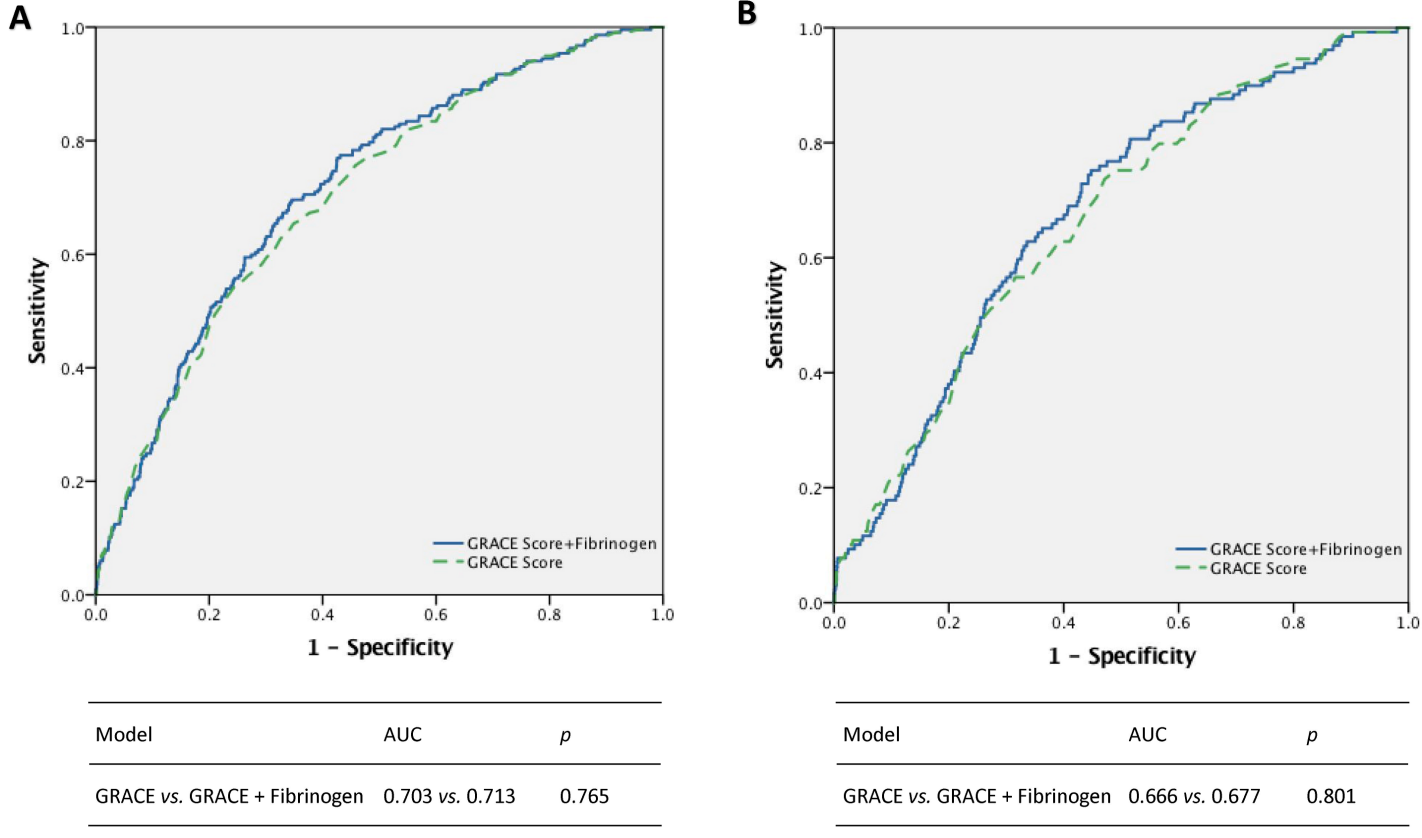
Incremental effects of fibrinogen in addition to GRACE risk score for all-cause mortality (**panel A**) and cardiac mortality (**panel B**).

Figure 3 demonstrates that in the present study cohort, the GRACE score has good predictive value for all-cause mortality among patients with ACS (AUC = 0.703). However, incorporating elevated Fib level into the GRACE model did not significantly increase the predictive value of the GRACE score; for instance, AUC only increased from 0.703 to 0.713 (*p* = 0.765). Similar results were obtained in analyses of cardiovascular death.

## DISCUSSION

This study found that an increase in Fib level was independently correlated with death risk in contemporary Chinese patients with ACS, but Fib did not increase the prognostic value of the GRACE score in ACS.

Fib is a key factor in blood coagulation. In addition, similarly to CRP, Fib is an important inflammatory cytokine in acute inflammation. It is widely recognized that Fib can lead to the occurrence of atherosclerosis and cardiovascular diseases [3, 14]. A series of studies has demonstrated that Fib level is significantly correlated with both the occurrence of CHD and the severity of coronary artery disease [15, 16]. However, the role of Fib in the secondary prevention of CHD remains controversial. One prospective study included 719 patients with CHD confirmed by coronary angiography. The results, obtained with a mean follow-up time of 6.5 years, indicated that Fib level was independently correlated with cardiovascular death [4]. Clinical data from 1,473 patients with unstable angina and non-Q-wave myocardial infarction from the Thrombolysis in Myocardial Infarction (TIMI) IIIB trial were analysed, and the results demonstrated that Fib level at admission was correlated with both coronary artery events and poor clinical outcome [5]. A substudy of the Fragmin and Fast Revascularization during Instability in Coronary Artery Disease (FRISC) trial indicated that in patients with unstable angina pectoris, increased Fib level was positively correlated with worse outcome; in addition, the increased risk associated with elevated Fib level was independent of the prognostic influence of myocardial damage [17]. Another study that included 515 consecutive patients with non-ST-segment elevation ACS revealed a correlation between Fib level and cardiac death that did not persist after correcting for multiple factors [18]. Recently, a consecutive study of 428 patients with STEMI also demonstrated that Fib concentrations were not particularly prognostic for major adverse cardiac events (MACEs) [19]. The following lines of reasoning may explain the aforementioned inconsistent results. ACS is a clinical syndrome with high heterogeneity, and patients with ACS exhibit markedly different disease severities and prognoses. Patients with various types of ACS might be recruited into different investigations, leading to a difference in patients’ disease severities across studies. Thus, the relationship between Fib level and prognosis was not consistent across different studies. Moreover, in the prior two decades, the application of reperfusion therapy and evidence-based medicine (EBM) has greatly improved the prognoses of patients with ACS. Therefore, studies conducted at different times may also have produced inconsistent results. The results of the current study indicated that Fib level at admission was independently correlated with death risk among contemporary Chinese patients with ACS, suggesting that Fib may play a pivotal role in the prognoses of such patients.

The GRACE score is a widely used tool in clinical practice for risk evaluation; however, this metric only considers two biomarkers, creatinine and troponin [9, 11]. Researchers have sought to introduce other biomarkers into the GRACE score system. These new biomarkers reflect other pathophysiological aspects of ACS and could provide additional information to improve the discrimination provided by the GRACE score. Several recent reports have indicated that CRP and neutrophil count could enhance the GRACE score's capacity to predict the prognoses of patients with ACS [12, 13]. Fib is similar to CRP, which is an inflammatory cytokine and has been independently correlated with cardiovascular events [3, 17]. Thus, it was assumed that the incorporation of Fib into the GRACE risk model would improve this model's ability to identify high-risk patients with ACS. However, the results of current study reveal that Fib level did not significantly enhance the predictive value of the GRACE score, although it was nonetheless independently associated with mortality in ACS. The GRACE score reflects a model established for risk assessment based on registry studies involving multiple centres and large samples [20, 21]. Many biomarkers are not included in the GRACE score, although these biomarkers may nonetheless affect the prognoses of patients with ACS. The reason why these biomarkers were not included in the GRACE scoring system is that they played minor roles in the risk model relative to the GRACE factors, such as age, renal function and myocardial damage [11]. Data from the AtheroGene study, which included 1806 patients with stable angina pectoris, indicated that despite the predictive value of Fib level for future cardiovascular risk, the incorporation of Fib level into a risk model based on traditional risk factors did not improve prognostic predictions [22]. In accordance with these results, a similar conclusion was obtained by another large-scale study of 13,195 patients with CHD [21]. The results of the current study further confirmed that the incorporation of Fib level did not enhance the prognostic value of the GRACE score in patients with ACS.

The present study had certain limitations. First, it is difficult to completely avoid the selection bias and confounding factors found in a registry study. Second, the sample for a single-centre study is subject to geographical restrictions that affect its representativeness. Third, as an observational study, the present study only indicated a correlation between Fib level and prognosis among patients with CHD. However, no conclusions regarding causal relationships could be drawn from this study. Finally, due to the low rate of cardiac mortality, the significance level of statistics would be influenced by the lack of power in the analysis of cardiac outcomes. In summary, we must regard this study's results with caution. We hope that in the future, a prospective multicentre study that could generate higher-quality results and thereby provide more evidence will be performed.

## CONCLUSIONS

Fib level at admission was independently associated with death risk among Chinese patients with ACS. However, the incorporation of Fib level at admission into the GRACE score did not improve this score's predictive value for death risk among these patients.

## MATERIALS AND METHODS

### Study population

The data source for this investigation was the West China Hospital CAD database. This single center database prospectively includes all the CAD or high risk patients undergoing angiography in West China Hospital affiliated to Sichuan University. For this analysis, we enrolled consecutive patients with CAD from January 2009 to September 2012 of the database. Patients with ACS were eligible for inclusion if they were restricted to participants with (1) angiographic evidence of ≥50% stenosis in ≥1 coronary vessels; and (2) ischemic chest discomfort that increased or occurred at rest; and/or (3) electrocardiography or cardiac biomarker criteria consistent with ACS. The exclusion criteria included malignancies, pregnancy, end stage renal disease (ESRD) and severe liver or hematological diseases. These inclusion and exclusion criteria were met by 2484 continuously CAD patients enrolled from database. After excluding patients with loss of follow-up (n = 192) and incomplete follow-up data (n = 39), 2253 patients were included in the data analysis. The study protocol was approved by the local institutional review boards in accordance with the Declaration of Helsinki. All subjects provided written informed consent before enrolment.

### Baseline characteristics

Demographic data, medical history, cardiovascular risk factor, vital signs at admission, medication at discharge, and final diagnosis were obtained from the patients’ electronic medical records and reviewed by a trained study coordinator. Blood sample were collected at admission and before angiography, and plasma biomarkers including Fib, liver and kidney function, blood glucose, serum lipid, etc. were analyzed in the department of Laboratory Medicine, West China hospital, accredited by the College of American Pathologists. Fib is assayed by Clauss method by the automatic coagulometer (Symex CA-7000, Japan). The GRACE risk prediction tool we used for analysis mortality has already been described previously [11]. The calculation of the GRACE risk score was performed using a computer program (http://www.outcomes-umassmed.org/grace). Hypertension was defined as those with systolic blood pressure (SBP) ≥ 140 mm Hg and/or diastolic blood pressure (DBP) ≥ 90 mm Hg and/or those receiving antihypertensive medications. Diabetes mellitus (DM) was diagnosed in patients who had previously undergone dietary treatment for diabetes, had received additional oral antidiabetic or insulin medication or had a current fasting blood glucose level of ≥7.0 mmol/L or random blood glucose level ≥11.1 mmol/L. Patients received care according to the usual practice; treatment was not affected by participation in this study.

### Follow-up and end points

The follow-up period ended on January 2013. Follow-up information was collected through contact with patients’ physicians, patients or their family. All data were corroborated with the hospital records. The primary end points in this study were all-cause mortality and the secondary end points were cardiovascular death, as documented in the database. Death was considered cardiac when it was caused by acute MI, significant arrhythmias, or refractory heart failure. Sudden unexpected death occurring without another explanation was included as cardiovascular death.

### Statistical analyses

We conducted the post-hoc analysis on a retrospective basis. Baseline demographics and clinical characteristics were compared between died patients and survivors. Continuous variables are expressed as the mean ± standard deviation (SD), and categorical variables are reported as counts and percentages. Analysis of *t* test and chi-squared tests were used to test for differences between groups for continuous and categorical variables, respectively. All patients were grouped into 3 categories by the tertiles of admission plasma Fib levels. Kaplan-Meier survival curves of the Fib tertile groups in relation to all-cause mortality and cardiac mortality in ACS patients were constructed and examined using the log-rank test for comparison. Hazard ratios (HRs) and 95 % confidence intervals (CIs) were calculated based on Cox proportional hazards regression models. The adjusted factors included age, sex, medical history (pre-hypertension and pre-diabetes mellitus), admission examination (systolic blood pressure, diastolic blood pressure, and heart rate), heart function (Killip level), admission lab test (blood glucose, total cholesterol, and serum creatinine), severity of CAD (left main artery and three vessel diseases), discharge medication (aspirin, clopidogrel, statin, ACE inhibitors or ARBs and beta-receptor blockers). Cox proportional hazards regression models were also used to investigate the independent effect of Fib and traditional risk factors on all-cause and cardiac mortality. To assess the predictive ability of the models, we considered the endpoints as a binary variable, and logistic regression was performed. Associated receiver operating characteristic (ROC) curves for predicted probabilities were drawn for GRACE score model and the GRACE score model added Fib. Two-sided p values of less than 0.05 indicated statistical significance. All analyses were performed with SPSS software (version 19.0).

## References

[1] Stefanadi E, Tousoulis D, Papageorgiou N, Briasoulis A, Stefanadis C (2010). Inflammatory biomarkers predicting events in atherosclerosis. Curr Med Chem.

[2] Fibrinogen Studies C, Danesh J, Lewington S, Thompson SG, Lowe GD, Collins R, Kostis JB, Wilson AC, Folsom AR, Wu K, Benderly M, Goldbourt U, Willeit J (2005). Plasma fibrinogen level and the risk of major cardiovascular diseases and nonvascular mortality: an individual participant meta-analysis. JAMA.

[3] Emerging Risk Factors C, Kaptoge S, Di Angelantonio E, Pennells L, Wood AM, White IR, Gao P, Walker M, Thompson A, Sarwar N, Caslake M, Butterworth AS, Amouyel P (2012). C-reactive protein, fibrinogen, and cardiovascular disease prediction. N Engl J Med.

[4] Espinola-Klein C, Rupprecht HJ, Bickel C, Lackner K, Schnabel R, Munzel T, Blankenberg S, AtheroGene I (2007). Inflammation, atherosclerotic burden and cardiovascular prognosis. Atherosclerosis.

[5] Becker RC, Cannon CP, Bovill EG, Tracy RP, Thompson B, Knatterud GL, Randall A, Braunwald B (1996). Prognostic value of plasma fibrinogen concentration in patients with unstable angina and non-Q-wave myocardial infarction (TIMI IIIB Trial). Am J Cardiol.

[6] O’Gara PT, Kushner FG, Ascheim DD, Casey DE, Chung MK, de Lemos JA, Ettinger SM, Fang JC, Fesmire FM, Franklin BA, Granger CB, Krumholz HM, Linderbaum JA (2013). 2013 ACCF/AHA guideline for the management of ST-elevation myocardial infarction: a report of the American College of Cardiology Foundation/American Heart Association Task Force on Practice Guidelines. Circulation.

[7] Amsterdam EA, Wenger NK, Brindis RG, Casey DE, Ganiats TG, Holmes DR, Jaffe AS, Jneid H, Kelly RF, Kontos MC, Levine GN, Liebson PR, Mukherjee D (2014). 2014 AHA/ACC guideline for the management of patients with non-ST-elevation acute coronary syndromes: a report of the American College of Cardiology/American Heart Association Task Force on Practice Guidelines. Circulation.

[8] Elbarouni B, Goodman SG, Yan RT, Welsh RC, Kornder JM, Deyoung JP, Wong GC, Rose B, Grondin FR, Gallo R, Tan M, Casanova A, Eagle KA (2009). Validation of the Global Registry of Acute Coronary Event (GRACE) risk score for in-hospital mortality in patients with acute coronary syndrome in Canada. Am Heart J.

[9] Eagle KA, Lim MJ, Dabbous OH, Pieper KS, Goldberg RJ, Van de Werf F, Goodman SG, Granger CB, Steg PG, Gore JM, Budaj A, Avezum A, Flather MD (2004). A validated prediction model for all forms of acute coronary syndrome: estimating the risk of 6-month postdischarge death in an international registry. JAMA.

[10] Tang EW, Wong CK, Herbison P (2007). Global Registry of Acute Coronary Events (GRACE) hospital discharge risk score accurately predicts long-term mortality post acute coronary syndrome. Am Heart J.

[11] Granger CB, Goldberg RJ, Dabbous O, Pieper KS, Eagle KA, Cannon CP, Van De Werf F, Avezum A, Goodman SG, Flather MD (2003). Fox KA and Global Registry of Acute Coronary Events I. Predictors of hospital mortality in the global registry of acute coronary events. Arch Intern Med.

[12] Zhang S, Wan Z, Zhang Y, Fan Y, Gu W, Li F, Meng L, Zeng X, Han D, Li X (2015). Neutrophil count improves the GRACE risk score prediction of clinical outcomes in patients with ST-elevation myocardial infarction. Atherosclerosis.

[13] Raposeiras-Roubin S, C Barreiro Pardal, Rodino Janeiro B, Abu-Assi E, Garcia-Acuna JM, Gonzalez-Juanatey JR (2012). High-sensitivity C-reactive protein is a predictor of in-hospital cardiac events in acute myocardial infarction independently of GRACE risk score. Angiology.

[14] Kannel WB, Wolf PA, Castelli WP, D’Agostino RB (1987). Fibrinogen and risk of cardiovascular disease. The Framingham Study. JAMA.

[15] Green D, Chan C, Kang J, Liu K, Schreiner P, Jenny NS, Tracy RP (2010). Longitudinal assessment of fibrinogen in relation to subclinical cardiovascular disease: the CARDIA study. J Thromb Haemost.

[16] Bolibar I, Kienast J, Thompson SG, Matthias R, Niessner H, Fechtrup C (1993). Relation of fibrinogen to presence and severity of coronary artery disease is independent of other coexisting heart disease. The ECAT Angina Pectoris Study Group. Am Heart J.

[17] Toss H, Lindahl B, Siegbahn A, Wallentin L (1997). Prognostic influence of increased fibrinogen and C-reactive protein levels in unstable coronary artery disease. FRISC Study Group. Fragmin during Instability in Coronary Artery Disease. Circulation.

[18] Bodi V, Sanchis J, Llacer A, Facila L, Nunez J, Bertomeu V, Pellicer M, Chorro FJ (2005). Risk stratification in non-ST elevation acute coronary syndromes: predictive power of troponin I, C-reactive protein, fibrinogen and homocysteine. Int J Cardiol.

[19] Ferraro S, Santagostino M, Marano G, Colli E, Vendramin C, Maffe S, Rossi L, Galvani M, Panteghini M, Bongo AS (2012). The prognostic value of plasma fibrinogen concentrations of patients with ST-elevation myocardial infarction and treated by primary percutaneous coronary intervention: a cautionary message. Scand J Clin Lab Invest.

[20] Investigators G (2001). Rationale and design of the GRACE (Global Registry of Acute Coronary Events) Project: a multinational registry of patients hospitalized with acute coronary syndromes. Am Heart J.

[21] Ndrepepa G, Braun S, King L, Fusaro M, Keta D, Cassese S, Tada T, Schomig A, Kastrati A (2013). Relation of fibrinogen level with cardiovascular events in patients with coronary artery disease. Am J Cardiol.

[22] Sinning JM, Bickel C, Messow CM, Schnabel R, Lubos E, Rupprecht HJ, Espinola-Klein C, Lackner KJ, Tiret L, Munzel T, Blankenberg S, AtheroGene I (2006). Impact of C-reactive protein and fibrinogen on cardiovascular prognosis in patients with stable angina pectoris: the AtheroGene study. Eur Heart J.

